# Supplementary figures and images for: Fungal feature tracker (FFT): A tool for quantitatively characterizing the morphology and growth of filamentous fungi
Source: PLoS Comput Biol. 2019 Oct 31;15(10):e1007428. doi: 10.1371/journal.pcbi.1007428 (PMC6822706; doi:10.1371/journal.pcbi.1007428)

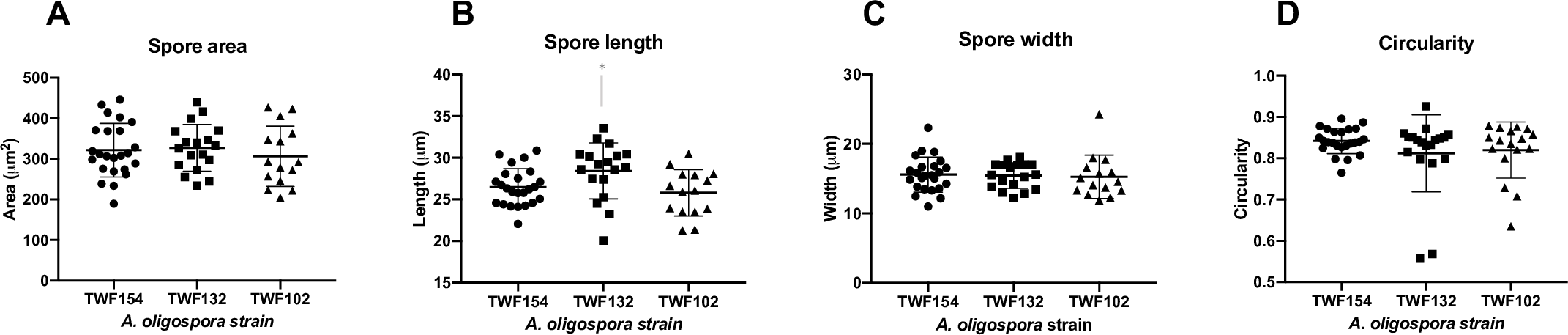

Supplement: S1 Fig — Comparison of conidial morphology for three A. oligospora strains. Spore area (A), length (B), width (C) and circularity (D), as computed by FFT using a total of 10 images per fungal strain. (TIF) [file pcbi.1007428.s002.tif]

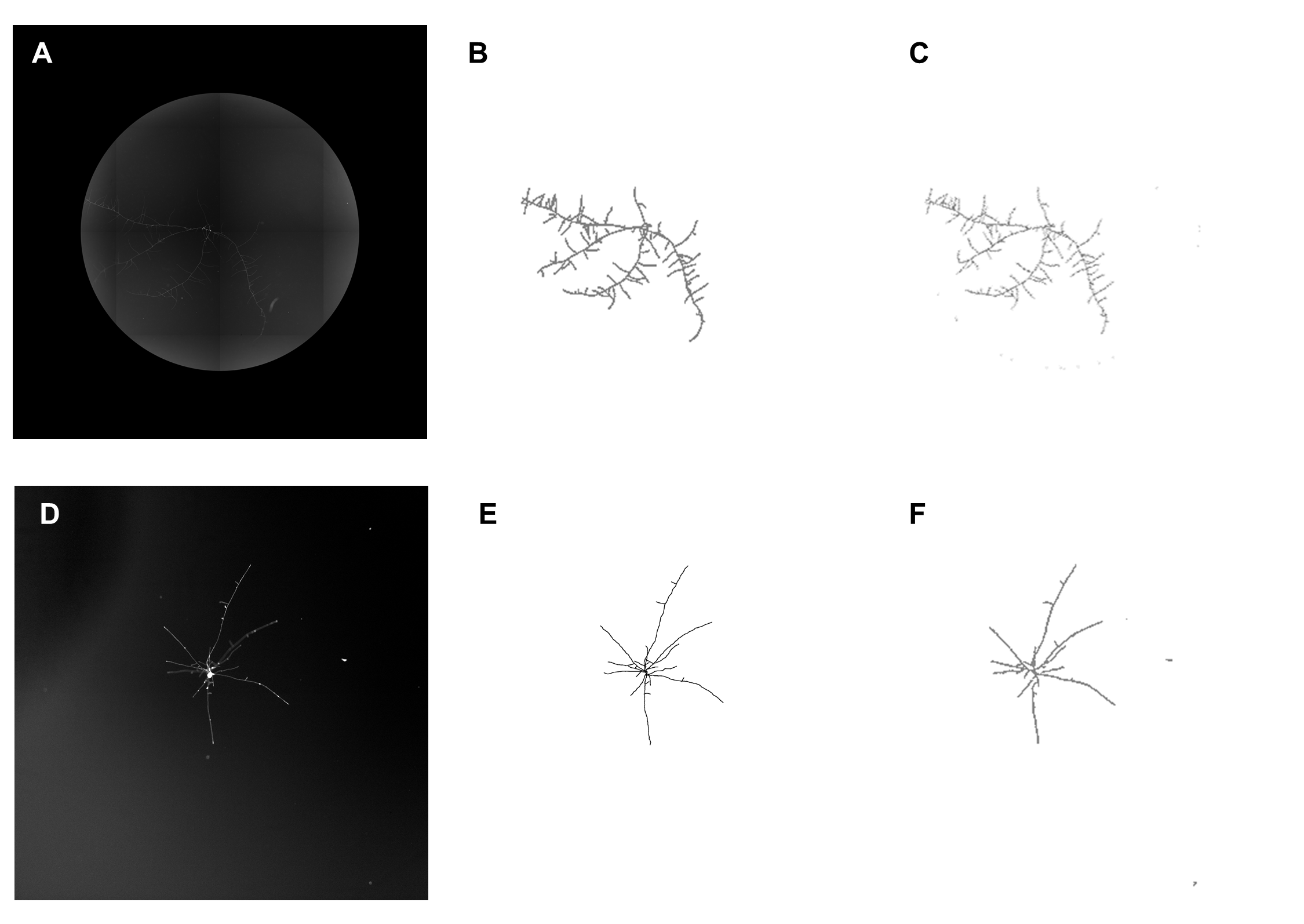

Supplement: S2 Fig — Original image 9 following mask subtraction (A), ground truth computed manually from the original image (B), and the image obtained by FFT after applying filters and binarization (C). Original image (D), ground truth image (E) and FFT-detected image (F) from a zoomed in view of image 4 showing an early-stage mycelium of A. oligospora. (TIF) [file pcbi.1007428.s003.tif]
