# Supplementary material for: Fungal feature tracker (FFT): A tool for quantitatively characterizing the morphology and growth of filamentous fungi
Source: PLoS Comput Biol. 2019 Oct 31;15(10):e1007428. doi: 10.1371/journal.pcbi.1007428 (PMC6822706; doi:10.1371/journal.pcbi.1007428)
Supplement: S1 Table — P-values obtain for each measure and pair of strains computed from the mean of six replicates per strain and time-point. (DOCX) [file pcbi.1007428.s004.docx]

| **Fungal Strains** | **Total number of tips** | **Total length** | **Area covered by the mycelium** |
| --- | --- | --- | --- |
| TWF102 vs TWF132 | 0.0003 | 0.0009 | 0.18 |
| TWF102 vs TWF154 | 0.0081 | 0.0049 | 1.07E-06 |
| TWF132 vs TWF154 | 1.65E-08 | 6.30E-08 | 1.40E-09 |
