# Supplementary material for: Fungal feature tracker (FFT): A tool for quantitatively characterizing the morphology and growth of filamentous fungi
Source: PLoS Comput Biol. 2019 Oct 31;15(10):e1007428. doi: 10.1371/journal.pcbi.1007428 (PMC6822706; doi:10.1371/journal.pcbi.1007428)
Supplement: S2 Table — P-values obtain for each measure, media condition and species combination computed from the mean of two replicates per species and time-point. (DOCX) [file pcbi.1007428.s005.docx]

| **Species and Media** | **Total number of tips** | **Total length** | **Area covered by the mycelium** |
| --- | --- | --- | --- |
| T.R vs N.C LNM | 0.25 | 0.039 | 0.0005 |
| T.R vs A.O LNM | 0.5 | 0.112 | 0.1418 |
| N.C vs A.O LNM | 0.072 | 0.64 | 0.0185 |
| T.R vs N.C PDA | 0.0458 | 0.1562 | 0.0134 |
| T.R vs A.O PDA | 0.0075 | 0.1015 | 0.0181 |
| N.C vs A.O PDA | 2.85E-08 | 8.24E-06 | 2.25E-06 |
